# Correction: A 3-D NanoMagnetoElectrokinetic model for ultra-high precision assembly of ferromagnetic NWs using magnetic-field assisted dielectrophoresis

**DOI:** 10.1039/d0ra90121k

**Published:** 2020-11-20

**Authors:** Sachin K. Singh, Md Mahadi Rajib, Justine L. Drobitch, Jayasimha Atulasimha, Supriyo Bandyopadhyay, Arunkumar Subramanian

**Affiliations:** Department of Mechanical and Industrial Engineering, University of Illinois at Chicago Chicago IL 60607 USA sarun@uic.edu; Department of Mechanical and Nuclear Engineering, Virginia Commonwealth University Richmond VA 23284 USA; Department of Electrical and Computer Engineering, Virginia Commonwealth University Richmond VA 23284 USA

## Abstract

Correction for ‘A 3-D NanoMagnetoElectrokinetic model for ultra-high precision assembly of ferromagnetic NWs using magnetic-field assisted dielectrophoresis’ by Sachin K. Singh *et al.*, *RSC Adv.*, 2020, **10**, 39763–39770, DOI: 10.1039/D0RA08381J.

The author regrets that the funding information was incorrectly shown in the acknowledgements section of the original manuscript. The corrected funding acknowledgement is as shown below.

All authors acknowledge support for this work, in part, from the National Science Foundation under Grant No. 1609303. A. S. and S. K. S. also acknowledge support, in part, from the National Science Foundation under Grant No. 1655496 and 1661038. The electrokinetics module used in the presented NanoMagnetoElectrokinetic modeling framework was developed with support from these grants.

The Royal Society of Chemistry apologises for these errors and any consequent inconvenience to authors and readers.

## Supplementary Material

